# Role of the Lateral Habenula in Pain-Associated Depression

**DOI:** 10.3389/fnbeh.2017.00031

**Published:** 2017-02-21

**Authors:** Yanhui Li, Yumeng Wang, Chengluan Xuan, Yang Li, Lianhua Piao, Jicheng Li, Hua Zhao

**Affiliations:** ^1^Department of Physiology, College of Basic Medical Sciences, Jilin UniversityChangchun, China; ^2^Department of Anesthesia, Neuroscience Research Center, First Hospital of Jilin UniversityChangchun, China

**Keywords:** chronic constriction injury, depression, lateral habenula, dorsal raphe nucleus, serotonin, chronic neuropathic pain

## Abstract

Patients with chronic pain have significantly higher incidences of depression and anxiety than the average person. However, the mechanism underlying this link has not been elucidated in terms of how chronic pain causes significant mood changes and further develops into severe anxiety or depression. The serotonergic system in the raphe nuclei is an important component in both pain processing and the pathogenesis of depression. Since the lateral habenular nucleus (LHb) controls the raphe nuclei, it may participate in the regulation of pain-associated depression. Thus, the aim of the current study was to investigate the role of the LHb in this pathophysiological process. We used chronic constriction injury (CCI) of the sciatic nerve in rats as a model for neuropathic pain and assessed the changes potentially related to the mood disorders. The forced swim test (FST) and sucrose preference test (SPT) were performed to determine the behavioral changes 28 days after pain surgery. Expression of β calmodulin-dependent protein kinase type II (βCaMKII) in the LHb, cytochrome-c oxidase (COX) activity in the LHb and dorsal raphe nucleus (DRN) and serotonin (5-HT) levels in the DRN were measured. We found an increasing in LHb activity and βCaMKII expression, and a decrease in neuronal activity in the DRN and 5-hydroxyindoleacetic acid (5-HIAA)/5-HT ratios in the CCI rats. These effects were accompanied by the depression-like behaviors. Lesions in the LHb improved the pain threshold and depression-like behavior in the rats. These results suggest that the LHb may play a role in pain-associated depression by affecting the activity of 5-HT neurons in the DRN. Furthermore, we showed that increases in the LHb-DRN pathway activity were a common neurobiological mechanisms for pain and depression, which may explain the coexistence of pain and depression.

## Introduction

According to clinical surveys, depression and anxiety incidence in patients with chronic pain is 3–5 times higher than in the average person (McWilliams et al., 2003; Beesdo et al., 2010). As the characteristic manifestation of chronic pain, negative emotions can also further exacerbate the feeling of pain, which is an important factor in determining the development of pain and outcome of the disease (Bair et al., 2003). However, the mechanism of how chronic pain develops into severe anxiety or depression is not well understood.

Numerous studies have demonstrated that some regions of brain are involved in both pain and emotion regulation (Bair et al., 2003; Robinson et al., 2009). Studies using magnetic resonance imaging (MRI) demonstrate that limbic brain regions, such as the insular cortex, medial prefrontal cortex, anterior cingulate cortex (ACC), amygdala, hippocampus and habenula (Hb) are activated or have abnormal changes not only in chronic pain patients, but also in depressive patients (Robinson et al., 2009; Sacher et al., 2012; Fomberstein et al., 2013; Boulos et al., 2017). It has been suggested that the functional changes of these brain areas may play a role in chronic pain associated with anxiety and depression symptoms.

The Hb, especially the lateral habenular nucleus (LHb), has attracted great attention in recent years due to its important role in depression and chronic pain (Hikosaka, 2010; Shelton et al., 2012a; Shen et al., 2012; Zhao et al., 2015). In rat models of depression, the activity of the LHb is increased. In addition, lesions in the LHb improves depression-like behaviors in rats by increasing the levels of serotonin (5-HT) in the dorsal raphe nucleus (DRN; Yang et al., 2008), since reduced levels of 5-HT in the DRN play an important role in the pathogenesis of depression (Stockmeier, 2003; Oquendo et al., 2007; Gao et al., 2008). In clinical practice, the high-frequency stimulation has been used in depressed patients to inhibit habenular function and thus achieve relieved depressive symptoms (Sartorius et al., 2010).

The serotonergic system in the raphe nuclei not only plays a role in the pathogenesis of depression, but also participates in pain processing. Descending fibers that initiate from 5-HT neurons in the raphe magnus nucleus (RMg) and DRN can project to the spinal cord and suppress incoming pain information (Stamford, 1995). Since the LHb controls the raphe nuclei, these findings provide evidence to support a potential role of the LHb in pain modulation. Additionally, the percentage of c-Fos–positive cells in the LHb was shown to increase in the rats receiving pain stimulation (Lehner et al., 2004; Li et al., 2016). Noxious stimuli could increase the LHb input to the rostromedial tegmental nucleus (RMTg), which projects to the monoamine system and inhibits its functions (Jhou et al., 2009; Lavezzi et al., 2012; Stamatakis and Stuber, 2012). It is also reported that some analgesic substance, such as the morphine, could produce analgesic effect by inhibiting activity of LHb neurons (Ma et al., 1992; Margolis and Fields, 2016). Overall, it appears that the LHb exhibits the same pathological changes in response to both depression and pain. In addition, the LHb has close functional and morphological relationships with central brain areas that are involved in pain and emotional response processing (Lecourtier and Kelly, 2007; Shelton et al., 2012b; Boulos et al., 2017). However, the functional changes linking the LHb to the process of chronic pain leading to depression have not been investigated, and thus we conducted the current study to address this issue.

## Materials and Methods

### Animals

The male Wistar rats were provided by the Department of Experimental Animals, Jilin University, Changchun, China, with the body weight of 180–200 g at the beginning of the experiments. The rats were housed in the standard conditions under a 12 h light-dark cycle (light on from 7 a.m. to 7 p.m.), with access to food and water *ad libitum*. All the animal care and use procedures were conducted following the Guide for the Care and Use of Laboratory Animals, and were approved by the Jilin University Animal Care Research Committee.

### Neuropathic Pain Model

Chronic constriction injury (CCI) was generated as previously described (Bennett and Xie, 1988). Rats were anesthetized by an intraperitoneal (i.p.) injection of 10% chloral hydrate, at a dose of 0.40 ml/100 g. The right sciatic nerve was exposed at the mid-thigh level, proximal to the sciatic trifurcation and four chromic gut (4/0), and ligatures were tied loosely around the nerve, 1.0–1.5 mm apart, so as not to compromise the vascular supply. Sham rats were treated in the same manner, but without nerve ligation.

### Nociceptive Behavioral Tests

The pain threshold in rats was tested at several time-points: before surgery, and 7 (CCI-7d), 14 (CCI-14d) and 28 (CCI-28d) days after surgery, as well as 7 days after the LHb was lesioned. The von Frey test was used for evaluating mechanical allodynia. Increasing force (range: 0–26 g) was applied over a period of 20 s. We took 26 g as the cutting force in the von frey test Thus the threshold was recorded as 26 g, when the force exceeded 26 g.

### Modified Forced Swimming Test

Rats were placed individually into a cylinder (20-cm in internal diameter, 50 cm in height) filled with water 30 cm in depth at 24 ± 1°C. A 15-min pre-test was performed 24 h before the 5-min test. The immobility or climbing time was monitored by a video camera and the behavior was blindly scored. The immobility time was determined by measuring the time when no additional activity was observed other than the movements necessary to keep the animal’s head above water. The climbing duration was measured during the time when rats made vigorous upward movements with their forepaws in and out of the water. Depressive-like behavior was defined as an increase in the immobility time and a decrease in the climbing time (Porsolt et al., 1978).

### Sucrose Preference Test

The animals were pre-exposed to two bottles of 1% sucrose solution (w/v) for 72 h at the beginning of the experiment. After that, 1% sucrose solution was replaced with water for 24 h. During the last 24 h, two bottles were randomly placed in the cage, 1% sucrose solution in one bottle and water in the other, and they were switched after 12 h to eliminate side preference. The amount of water and sucrose consumed during the 24 h was measured and the percent preference for sucrose consumption was calculated as a percentage of sucrose solution consumed in relation to the total fluid intake.

### Locomotor Activity

Free exploratory ambulation was recorded individually for 5 min in a transparent box (104 × 104 × 40 cm) using a spontaneous motor activity recording and tracking video system (Noldus, Holland) in a light-attenuated room. The total distance traveled was measured as an indicator of locomotor activity. After the test, rats were returned to their home cages and the box was cleaned with 70% ethanol between tests.

### Quantitative Cytochrome-C Oxidase Histochemistry

An established method for detecting quantitative cytochrome-c oxidase (COX) activity has been previously described (Gonzalez-Lima and Cada, 1994). Briefly, after being anesthetized, the brains were quickly extracted and frozen in −80°C isopentane for 3 min. Thirty-micrometer-thick coronal brain sections were obtained using a cryostat microtome (CM1950, Leica) and placed on slides. Sections were stored at −80°C. At the time of staining, sections were air-dried to room temperature for 5 min and then fixed in 0.5% glutaraldehyde containing 10% sucrose phosphate buffer. After rinsed three times (5-min each) in phosphate buffered saline (PBS), the slides were incubated in PBS containing 0.05% diaminobenzidine, 0.01% cytochrome-c (from horse heart) and 5% sucrose in the dark at room temperature. The sections were then dehydrated using ethanol, cleared using xylene, and coverslipped. To reduce the inter-batch variability, all sections were stained using the same batch. The optical density of the COX signal was analyzed using image analysis software (Image-Pro Plus, version 6, Media Cybernetics, Inc., Rockville, MD, USA). Three non-overlapping pictures were obtained bilaterally for each section. Optical density was obtained as the average values of the three sections for each rat.

### Western Blot

The brains were quickly extracted and LHb tissues were removed bilaterally. The tissue was collected and homogenized in RIPA lysis buffer containing 12 μg phenylmethylsulfonyl fluoride. Proteins were loaded onto 12% SDS-PAGE gels, electrophoretically separated, and transferred to polyvinylidene fluoride membranes for western blot analysis. The primary antibodies used were β calmodulin-dependent protein kinase type II (βCaMKII, ab34703; Abcam, Cambridge, UK) and anti-β-actin (bs-0061-R; Bioss Inc., Woburn, MA, USA). These antibodies were detected using horseradish peroxidase-conjugated goat anti-rabbit antibody (bs-0295-HRP; Bioss Inc., Woburn, MA, USA). High sensitivity electrochemiluminescence reagent was used to visualize the bands.

### High-Performance Liquid Chromatography Analysis

Rats were anesthetized using chloral hydrate (400 mg/kg, i.p.), and their brains were quickly extracted. Brain regions were dissected out using a vibratome. Samples were immediately frozen in liquid nitrogen and stored at −80°C until analysis.

Samples were homogenized in 200 ml of ice-cold 0.05 M perchloric acid and centrifuged at 14,000× g for 20 min. The supernatant was analyzed directly by high performance liquid chromatography (HPLC) using a fluorescence detector and a LUNA C18 column (150 mm × 4.6 mm inner diameter, 5 μm pore size). The mobile phase used for HPLC was 0.1 M sodium acetate, 0.1 mM disodium ethylenediaminetetraacetic acid, 10% methanol, pH 5.1. The excitation wavelength was 290 nm and the emission wavelength was 330 nm. Measurements were presented in ng per mg of tissue protein of the primary metabolite 5-hydroxyindoleacetic acid (5-HIAA) to that of 5-HT.

### LHb Lesion Surgery

Rats were anesthetized using chloral hydrate (400 mg/kg, i.p.) and positioned in an S-R stereotaxic instrument (Narishige Corporation, Japan) with the tooth bar 3.3 mm below the ear bars, according to the atlas of Paxinos and Watson (2008). Body temperature was maintained at 37°C using a heating pad. A bilateral electrolytic lesion of the LHb was performed using stainless steel electrodes (35 KΩ) and a DC current of 0.35 mA for 40 s was applied using an electronic stimulator (SEN-7130, Nihon Kohden Kogyo Co. Ltd., Tokyo, Japan). The coordinates for electrodes were as follows: 3.6–3.8 mm posterior to bregma, 0.8 mm lateral to midline, and 4.3–4.6 mm ventral to the dural surface. For the sham-treated animals, the electrodes were inserted at the same coordinates but without any current being passed. After the surgery, rats were placed in recovery chambers to allow them completely woke up before being returned to their home cages.

### Analysis of Lesions

**Figure 1:**
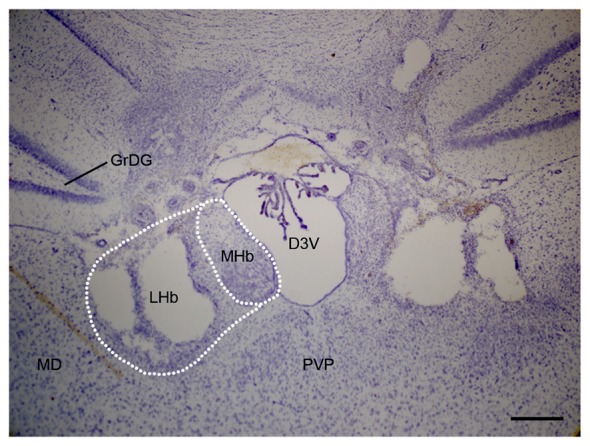
**Representative Nissl staining photomicrograph of bilateral LHb lesion in chronic constriction injury (CCI) rats.** Scale bars, 200 μm (LHb, lateral habenula; MHb, medial habenula; D3V, dorsal third ventricle; GrDG, granular layer dentate gyrus; MD, mediodorsal nucleus; PVP, posterior paraventricular nucleus).

Rats were deeply anesthetized using pentobarbital (80 mg/kg, i.p.) and were perfused with normal saline followed by 4% formaldehyde. Brains were cryoprotected and sectioned into 50-μm-thick slices. Sections were mounted and Nissl-stained. Damage to the LHb was assessed, with comparison to a reference atlas, by an observer who was blinded to the behavioral results (Figure 1).

### Statistical Analysis

All data were expressed as means ± standard errors of mean (SEM). Statistical analysis was performed using IBM SPSS statistical software (version 12.0.1, SPSS Inc., Chicago, IL, USA). Significance was set at *P* < 0.05. Repeated-measures ANOVA was used to assess the withdrawal threshold (group × time), and the Student’s *t*-test was used for all the other analyses.

## Results

### Establishment of the Chronic Pain Model

**Figure 2:**
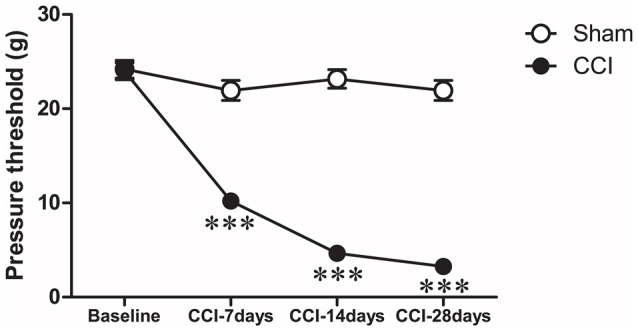
**Time-course changes in paw withdrawal threshold after CCI.** Ipsilateral paw withdrawal threshold (g) in response to paw-pressure stimulation: ***P < 0.001 vs. baseline (Day-1) by repeated measures, two-way ANOVA, followed by Tukey-Kramer honest significant difference post-test.

We used the von Frey test to examine the nociceptive thresholds of rats in both CCI and CCI-sham groups on day-1 (1 day before surgery), and 7, 14 and 28 days after surgery. We used a repeated measures two-way ANOVA (group × time) to test for the differences in the nociceptive threshold in these two groups of rats at different time-points (Figure 2). The results indicated a significant difference in the nociceptive threshold between the two groups (*F*_(3,156)_ = 179.8, *P* < 0.001). Significant differences were also found in the nociceptive threshold at different time-points within the same groups (*F*_(3,156)_ = 121.2, *P* < 0.001). Together these results indicated a decrease in the nociceptive threshold in the CCI group compared to the sham group, which validated that the pain model was successfully established.

### Changes in Behavioral Test after Neuropathic Pain Surgery

**Figure 3:**
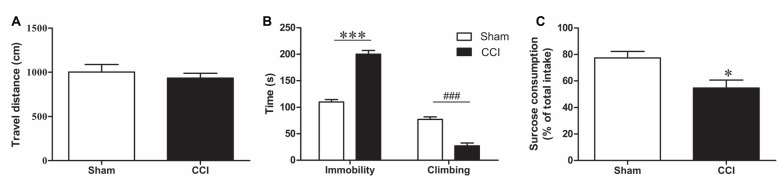
**Behavioral changes after CCI. (A)** Change in locomotor activity after CCI (*n* = 8). **(B)** The behavioral change in forced swimming test (FST) after CCI. Bar graphs show immobility time and climbing time in the FST of CCI rats and sham rats (*n* = 8). **(C)** The sucrose consumption in sucrose preference test (SPT) of CCI rats and sham rats (*n* = 8). Data are expressed as mean ± standard error of mean (SEM) in bar graphs, and were analyzed using an independent Student’s *t*-test (*P < 0.05, ***P < 0.001, ^###^*P* < 0.001).

To avoid an effect of CCI on the overall activity, which would compromise the result accuracy in the forced swimming test (FST), the total distance traveled was measured before the FST as an indicator of locomotor activity, and no significant difference was found between the groups (*p* > 0.05, Figure 3A).

The FST was used to evaluate depression-like behaviors in CCI model rats. After 28 days of CCI surgery, the immobility time was prolonged (*t* = 10.55, *P* < 0.001, Figure 3B) and climbing time were reduced in CCI rats compared to the sham-treated rats (*t* = 7.07, *P* < 0.001, Figure 3B).

In the SPT we found that the CCI rats had a lower sucrose consumption than the sham group 28 days after CCI surgery (*T* = 2.944, *P* < 0.05, Figure 3C). These results confirmed that CCI model rats exhibit obvious depression-like behavior.

### Change in COX Activity and βCaMKII Expression in the LHb of CCI Rats

**Figure 4:**
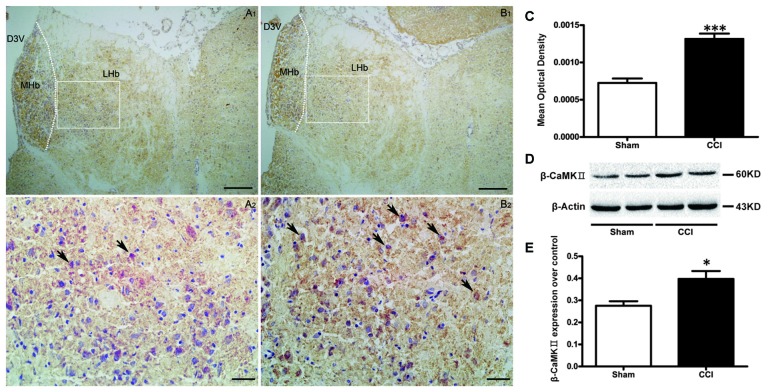
**The effect of CCI operation on LHb activity. (A,B)** Representative histochemistry images of cytochrome-c oxidase (COX)-positive cells in the LHb of the sham group (**A1,A2**; *n* = 10) and the CCI group (**B1,B2**; *n* = 10). Scale bars: 100 μm (**A1,B1**, ×100 magnification); 20 μm (**A2,B2**, ×400 magnification). **(C)** Change of the COX activity in the LHb of CCI rats and sham rats (*n* = 10). Data are expressed as mean ± SEM in bar graphs. **(D,E)** Western blotting results for β calmodulin-dependent protein kinase type II (β CaMKII) expression in the LHb of the CCI and sham (*n* = 9) groups, data are expressed as mean ± SEM in bar graphs (MHb, medial habenula; LHb, lateral habenula; D3V, dorsal third ventricle; *P < 0.05, ***P < 0.001 as assessed by an independent Student’s *t*-test).

COX activity in the LHb (Figures 4A–C), was increased in the CCI group, compared to sham-treated rats (*t* = 6.16, *P* < 0.001, Figure 4C).

Western blotting showed an up-regulated expression of βCaMKII in the CCI group compared to the sham group (*t* = 2.94, *P* < 0.05, Figures 4D,E), which suggests a hyperactivity of the LHb neurons present in CCI rats.

### Change in the Activity of DRN in the CCI Rats

#### Altered COX Activity in the DRN of CCI Rats

**Figure 5:**
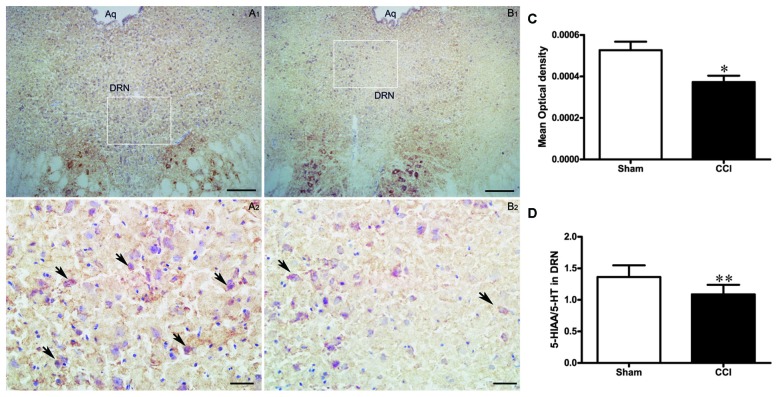
**The effect of CCI operation on DRN activity. (A,B)** Representative histochemistry images of COX-positive cell in the DRN of the sham group (**A1,A2**; *n* = 10) and the CCI group (**B1,B2**; *n* = 10). Scale bars: 100 μm (**A1,B1**, ×100 magnification); 20 μm (**A2,B2**, ×400 magnification). **(C)** Change of COX activity in the DRN of rats in the CCI and sham groups (*n* = 10). Data are expressed as mean ± SEM in bar graphs. **(D)** The ratio of 5-hydroxyindoleacetic acid (5-HIAA)/serotonin (5-HT) in the DRN of the CCI and sham groups (*n* = 8); data are expressed as mean ± SEM in bar graphs (DRN, dorsal raphe nucleus; Aq, aqueduct; *P < 0.05, **P < 0.01 as assessed by an independent Student’s *t*-test).

COX activity in the DRN of the CCI group was significantly decreased compared to the rats in the sham group (*t* = 3.0, *P* < 0.05, Figures 5A–C).

#### Change in 5-HT Level in the DRN of CCI Rats

To further investigate the effects of chronic pain on the activity of DRN neurons, we measured the 5-HT content and its metabolites in the DRN using HPLC and calculated the metabolites/neurotransmitter ratio (5-HIAA/5-HT) to represent the serotonergic activity in the DRN, i.e., increased ratio means a high level of activity in the DRN neurons. We found that the 5-HIAA/5-HT ratio was lower in the CCI group compared to the sham group (*t* = 3.24, *P* < 0.01, Figure 5D), suggesting that 5-HT function is reduced in the DRN of CCI rats.

### Changes in Neuropathic Pain Threshold and Depression Behavior in CCI Rats Following LHb Lesion

#### Change in Neuropathic Pain Threshold in CCI Rats after LHb Lesion

**Figure 6:**
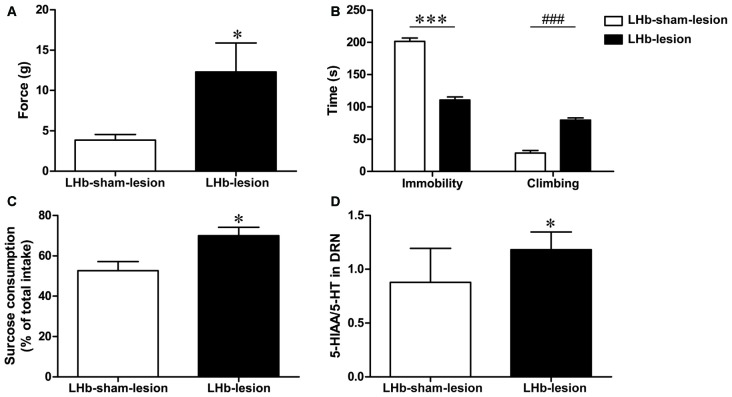
**The effect of LHb lesion on neuropathic pain threshold and depression behavior in CCI rats. (A)** The pressure threshold of LHb-lesion group and LHb-sham-lesion group in CCI rats (*n* = 8). **(B)** The duration of immobility and climbing time in the FST of LHb-lesion and LHb-sham-lesion groups (*n* = 8). **(C)** The sucrose consumption in SPT of LHb-lesion and LHb-sham-lesion groups (*n* = 8). **(D)** The ratio of 5-HIAA/5-HT in the DRN of the LHb-sham-lesion groups and the LHb-lesion group in CCI rats (*n* = 8). Data are expressed as mean ± SEM in bar graphs (*P < 0.05, ***P < 0.001, ^###^*P* < 0.001 as assessed by an independent Student’s *t*-test).

To further demonstrate the role of LHb in CCI rats, we examined the neuropathic pain threshold 7 days after the LHb lesion. The neuropathic pain threshold in the LHb-lesion group had a significant increase compared to the LHb-sham group (*t* = 2.29, *P* < 0.05, Figure 6A). This result suggests that LHb lesions may improve hyperalgesia in CCI rats.

#### Change in Depression Behaviors in CCI Rats after LHb Lesion

In this study, we used FST and SPT to determine the effect of LHb lesions on depression behaviors in CCI rats. The results showed that LHb-lesion group had a shorter immobility time (*t* = 12.76, *P* < 0.001, Figure 6B) and longer climbing time (*t* = 9.25, *P* < 0.001, Figure 6B) in the FST, compared with the LHb-sham-lesion group.

In the SPT, sucrose consumption in the LHb-lesion group was increased, compared to the LHb-sham-lesion group (*T* = 2.812, *P* < 0.05, Figure 6C). These results indicate that the LHb lesion could improve depression-like behaviors in CCI rats.

#### Change in 5-HT Level in DRN of CCI Rats after LHb Lesion

The levels of 5-HT in the DRN were decreased in CCI model rats as mentioned above. Furthermore, the 5-HIAA/5-HT ratio in the LHb-lesion group was higher than that in the LHb-sham-lesion group (*t* = 2.41, *P* < 0.05, Figure 6D). This indicates that lesions in the LHb may result in increased concentration of 5-HT in the DRN.

## Discussion

In this study, the chronic neuropathic pain model was produced using the classic method described by Bennett and Xie (1988). Rats were tested for allodynia and hyperalgesia at several time-points: before surgery (baseline) and 7 (CCI-7d), 14 (CCI-14d) and 28 (CCI-28d) days after surgery. Results showed that over time, compared to the sham group, the rats in the CCI group had significantly lower mechanical pain threshold, indicating the success in generating a chronic neuropathic pain model. Compared to other groups, the CCI group rats had the most pronounced change in pain threshold 28 days after surgery. Thus, this time-point was chosen for the behavioral tests. In the FST, the CCI model rats had significantly prolonged immobility times and shorter climbing times, as well as lower sucrose consumption in the SPT than the sham group. These results indicate the development of depression-like behavior in CCI rats. This is consistent with the study by Alba-Delgado et al. (2013) who reported that CCI model rats showed depression-like behavior 28 days after surgery, which was not obvious at earlier time points (7 or 14 days after the surgery). Significant depression-like behaviors has also been found in spinal nerve ligation neuropathic pain model rats 15 and 30 days after surgery (Suzuki et al., 2007). These animal studies suggest that chronic pain may cause depression-like disorders, which is consistent with the clinical observation that most patients with chronic pain also have comorbid depression (Beesdo et al., 2010).

5-HT neurons in the DRN play an important role in pain regulation (Wang and Nakai, 1994; Bobinski et al., 2015). In addition, the hypofunction of 5-HT neurons is considered to be an important causal factor of depression (Stockmeier, 2003; Oquendo et al., 2007). The LHb is a key component in the control of DRN and has a close relationship with pain regulation (Shelton et al., 2012a; Zhao et al., 2015; Margolis and Fields, 2016). However, it is still unclear whether the LHb plays a role in chronic neuropathic pain-related depression by affecting the DRN. In this study, we found increased LHb neuron activity in CCI model rats with severe depressive symptoms, 28 days after surgery. It was reported that a majority of LHb neurons (75%) recorded electrophysiologically was activated by peripheral noxious stimuli (Benabid and Jeaugey, 1989). Shelton et al. (2012b) found the activation of the LHb in people receiving noxious stimuli using high-field MRI technology (Shelton et al., 2012b). The results from both animal experiments and clinical observation in patients with pain have confirmed that the LHb can receive pain messages and is activated by pain.

The current study showed that CCI model rats with depressive behavior had significantly decreased COX activity in DRN neurons. The HPLC results showed that the 5-HIAA/5-HT ratio in the DRN was relatively reduced in the CCI model rats compared to sham-treated rats. Together, these findings suggest that 5-HT function is reduced in the DRN of CCI model rats.

Studies have shown that 5-HT neurons in the raphe nuclei, such as the RMg and the DRN are major components of the central analgesic system (Bardin, 2011). This system can inhibit afferent nociceptive stimuli by projecting efferent fibers to the spinal cord to achieve analgesic effects (Messing and Lytle, 1977; Wang and Nakai, 1994). The 5-HT neurons mainly in DRN are inhibited by activation of the LHb (Wang and Aghajanian, 1977; Varga et al., 2003). The LHb inhibits 5-HT neurons in the DRN by activating GABA interneurons, because the 5-HT neurons in the DRN have GABA receptors and these receptors are regulated by GABAergic neurons (Wang et al., 1992; Craige et al., 2015). The LHb also regulates the activity of DRN neurons indirectly by impacting the GABAergic neurons in the RMTg, which projects to and inhibits DRN 5-HT neurons (Lavezzi et al., 2012). The electrical stimulation of LHb can inhibit firing rate of 5-HT neurons in the DRN, as identified by electrophysiological characteristics. Antagonists of GABA receptor can block the inhibitory effect of the LHb on the 5-HT neurons in the DRN (Wang and Aghajanian, 1977). In the CCI model rats, enhanced LHb activity subsequently increased the inhibition of DRN 5-HT neurons. Since the 5-HT neurons in the DRN are known to reduce pain via descending pathway, the weakening of this effect can explain the decreased pain threshold in CCI model rats. The pain threshold and the 5-HIAA/5-HT ratio of CCI model rats increased after the LHb lesion, compared to sham-treated rats. These results indicate that the pain threshold can be increased in CCI model rats by increasing 5-HT function in the DRN by introducing a lesion to the LHb. More significantly, the combined depression behavior in the CCI model rats was improved by the LHb lesion.

Previous studies have shown that neuronal metabolism is increased in the LHb in four different animal models of depression. Specifically, in the congenitally learned helpless (cLH) rat model, LHb metabolism increased by 64%–71% (Caldecott-Hazard et al., 1988; Shumake et al., 2003). There is also evidence that LHb neuronal activity is increased in patients with depression (Aizawa et al., 2013). In animal models and patients with depression, the inhibition of the LHb can cause remission in both the rats’ depression-like behaviors and patients’ depressive symptoms (Yang et al., 2008; Sartorius et al., 2010). The mechanism by which a lesion in the LHb improves the behavioral response in depressed rats can be explained by the increased 5-HT levels in the DRN. In the current study, the increased activity of the LHb in CCI model rats offered a reasonable explanation as for how chronic neuropathic pain causes depressive behaviors. We therefore suggest that the enhanced activity of the LHb-DRN pathway may not only be involved in the pathogenesis of depression, but it also represent an important underlying mechanism for the development of chronic pain.

Li et al. (2013) found that βCaMKII expression is significantly increased in the LHb of cLH rats. βCaMKII overexpression in the LHb can induce depression-like behaviors in rats and increase miniature excitatory postsynaptic currents in the LHb. In the current study, we found that βCaMKII expression within the LHb was significantly increased in CCI model rats, suggesting that βCaMKII may also be an important molecule in the LHb involved chronic neuropathic pain.

In conclusion, the LHb and the DRN play important roles in the regulation of depression and pain. These two areas are closely related both anatomically and functionally (Wang and Aghajanian, 1977; Yang et al., 2014; Luo et al., 2015; Zhao et al., 2015). Our previous studies have shown that the LHb lesions can improve depression-like behaviors by increasing the level of 5-HT in the DRN (Yang et al., 2008). In the current study, using CCI model rats, we observed increased LHb activity and βCaMKII expression, and decreased DRN neuronal activity and the 5-HIAA/5-HT ratio. Of note, depression-like behaviors were also observed in these rats. Lesions in the LHb improved both the pain threshold and depression-like behaviors in CCI model rats. Together, our results suggest that increased activity of the LHb-DRN pathway may be a common neurobiological mechanism underlying pain and depression, which have provided explanation for their coexistence.

## Author Contributions

Design of the experiments: HZ and YanhuiL. Performance of the experiments: YanhuiL, YW, JL and LP. Collection and analysis of data: YanhuiL and CX. Draft of manuscripts and preparation of figures: YanhuiL, YangL and HZ. All the authors approved the final version of the manuscript.

## Funding

This study was supported by the National Natural Science Foundation of China (Grant Nos.: 91332117 and 81271465).

## Conflict of Interest Statement

The authors declare that the research was conducted in the absence of any commercial or financial relationships that could be construed as a potential conflict of interest.
